# Non-invasive detection of regulatory T cells with Raman spectroscopy

**DOI:** 10.1038/s41598-024-64536-0

**Published:** 2024-06-18

**Authors:** N. Pavillon, E. L. Lim, A. Tanaka, S. Hori, S. Sakaguchi, N. I. Smith

**Affiliations:** 1 Biophotonics Laboratory, Immunology Frontier Research Center (IFReC), Osaka University, Yamadaoka 3-1, Suita, Osaka 565-0871 Japan; 2 Experimental Immunology, Immunology Frontier Research Center (IFReC), Osaka University, Yamadaoka 3-1, Suita, Osaka 565-0871 Japan; 3 Department of Frontier Research in Tumor Immunology, Graduate School of Medicine, Osaka University, Yamadaoka 2-2, Suita, Osaka 565-0871 Japan; 4 Laboratory of Immunology and Microbiology, Graduate School of Pharmaceutical Sciences, The University of Tokyo, Bunkyo-ku, Hongo 7-3-1, Tokyo, 113-0033 Japan; 5 Laboratory of Experimental Immunology, Institute for Life and Medical Sciences, Kyoto University, Sakyo-ku, Shogoin Kawahara-cho 53, Kyoto, 606-8507 Japan; 6 Center for Infectious Disease Education and Research (CiDER), Osaka University, Yamadaoka 2-8, Suita, Osaka 565-0871 Japan; 7 Open and Transdisciplinary Research Institute (OTRI), Osaka University, Yamadaoka 3-1, Suita, Osaka 565-0871 Japan

**Keywords:** Raman spectroscopy, Lymphocyte differentiation, Bioinformatics, CD4-positive T cells

## Abstract

Regulatory T cells (Tregs) are a type of lymphocyte that is key to maintaining immunological self-tolerance, with great potential for therapeutic applications. A long-standing challenge in the study of Tregs is that the only way they can be unambiguously identified is by using invasive intracellular markers. Practically, the purification of live Tregs is often compromised by other cell types since only surrogate surface markers can be used. We present here a non-invasive method based on Raman spectroscopy that can detect live unaltered Tregs by coupling optical detection with machine learning implemented with regularized logistic regression. We demonstrate the validity of this approach first on murine cells expressing a surface Foxp3 reporter, and then on peripheral blood human T cells. By including methods to account for sample purity, we could generate reliable models that can identify Tregs with an accuracy higher than 80%, which is already comparable with typical sorting purities achievable with standard methods that use proxy surface markers. We could also demonstrate that it is possible to reliably detect Tregs in fully independent donors that are not part of the model training, a key milestone for practical applications.

## Introduction

Immune defenses are defined and regulated by complex sets of various cellular populations, each having specific functions both up- and down-regulating the immune responses. Among these, T cells are one of the key components of adaptive immunity, that provide defenses against pathogens, especially viruses, through the recognition of specific antigens^1^. T cells are commonly divided in two main sub-types, CD4^+^ T cells (denoted as conventional (Tconv)) and CD8^+^ T cells. In contrast to these two sub-types, regulatory T cells (Treg) are a small subset of CD4-expressing T cells specialized in suppressing unwanted immune responses (e.g. against self-tissues). Tregs have been found to be indispensable in maintaining immunological self-tolerance, thereby preventing autoimmune reactions and diseases^2–4^. Treg functions are mostly regulated by the transcription factor Foxp3, whose deficiency can lead to severe autoimmune disorders^5,6^, and several diseases have been linked to mutations of genes associated with Tregs functions^7,8^. Furthermore, Tregs are increasingly considered a highly promising clinical tool for the therapy of autoimmune diseases and other inflammatory conditions^9,10^.

The ability to isolate Treg populations is therefore of very high importance, but in practice is not trivial. They are predominantly defined by the intracellular expression of the transcription factor Foxp3^11,12^, which is considered one of the most specific marker of Tregs, although sorting Tregs is still challenging and requires the combination of multiple markers^13,14^. Furthermore, since it is expressed inside the cell, it is not practically possible to reliably detect Foxp3 in live unmodified cells. Instead, researchers rely on surrogate surface markers to identify Treg populations, such as interleukin (IL)-2 (CD25) or IL-7 (CD127) receptors^15,16^, or the glucocorticoid-induced tumor necrosis factor receptor (GITR)^17^. However, many of these surrogate markers are also upregulated by Tconv cells upon activation, potentially leading to contamination in the isolation process^18^. In addition to the purity issues in using surface markers to isolate Tregs, the most widely used high throughput approach uses fluorescent-activated cell sorting (FACS), which can affect cellular function through the high-density binding of antibody to immune-related receptors^19,20^. The development of non-invasive methods that can reliably detect and isolate functional live Treg populations is therefore of high relevance.

We describe here an approach based on optical detection that can identify cellular phenotypes from live unaltered cells, based on the measurement of the Raman spectrum. These signals originate from endogenous intracellular molecules but use only light to probe the inside of the cell^21^. The non-invasiveness and specificity of such an approach has led to various applications in biology, such as bacterial identification^22,23^, cancer detection^24,25^ or monitoring of cellular processes^26^. These spectral measurements have recently been combined with automated platforms to reach relatively high throughput for single-cell analyses^27,28^, and it has been shown that they can be employed to detect various immune cells phenotypes^29^. They have been used recently to study T cell activation^30^ and differentiation^31^.

Here we use this method combined with machine learning to non-invasively detect Treg subpopulations from CD4^+^ lymphocytes, and highlight the spectral differences that are present between Tconv and Treg cells. The implementation is based on regularized logistic regression through least absolute shrinkage and selection operator (LASSO). This method was selected as it provides highly stable models across varied data pools^27^, along with the fact that the resulting linear models enable direct interpretation of the contributing molecular species^32^. We first demonstrate the applicability of this method with murine splenocytes modified to have a surface Foxp3 reporter^33^, and then show that the same approach can be used for detecting human Tregs isolated from peripheral blood mononuclear cells (PBMC). We then show how the optically-derived results are highly correlated with Foxp3 expression, and further demonstrate that our models are also transferable to new donors, a key point for practical applicability of such an approach.

## Results

We employed a custom-made Raman system that was optimized for single-cell analysis^29^, used here to measure lymphocytes and discriminate Tconv and Tregs. The system was recently automated to ensure higher throughput and better reproducibility^31^.

### Raman can non-invasively distinguish Tconv and Treg

To first assess the capability of this approach to discriminate these two phenotypes, we used CD4^+^ cells purified from murine spleen by MACS negative selection, which were therefore unaltered by the sorting process. The CD4^+^ population was then FACS-sorted to separate Tconv and Treg cells by staining a Foxp3-hCD2 reporter located at the surface membrane^33^ that allows for easy separation of live Tconv and Treg cells (see sorting purity in Supplementary Fig. S1). A summary of the markers employed for sorting is provided in Fig. S2, and typical distributions after sorting are shown in Supplementary Fig. S3.

Raman measurements are then performed on the Tconv and Treg populations retrieved from FACS. The signal emitted by fluorescent dyes can have a very strong influence on the measurements, as Raman scattering is far weaker than typical fluorescent emissions. We therefore selected dyes whose excitation band is far from the Raman laser (532 nm) and that emit outside the Raman spectral window. The dyes employed in this study were individually confirmed as having no significant influence on the Raman signal compared to cell-to-cell variations^31^.

**Figure 1:**
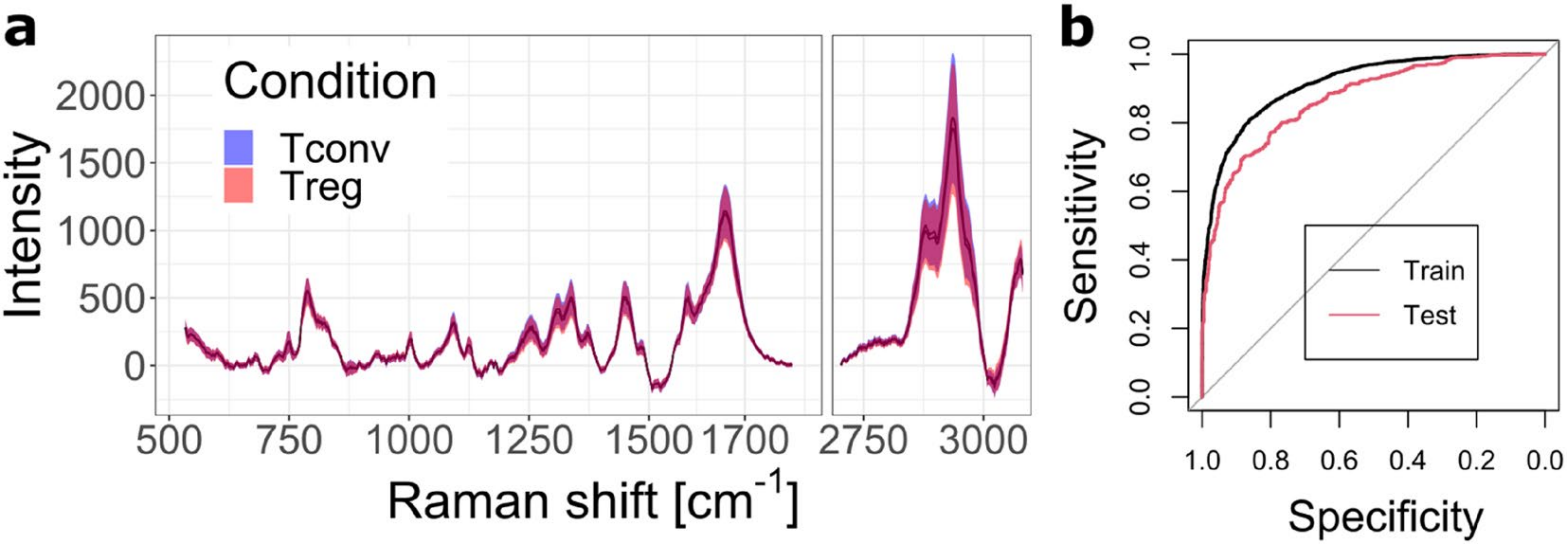
(**a**) Average Raman spectra for murine Tconv and Treg (N = 3113 and 3117, respectively, measured over 3 days), showing that few differences may be identified by eye, far below the intra-group variability. (**b**) ROC curves of classification models identifying Treg cells (20% of test data). Test area under the curve (AUC): 0.869.

As shown in Fig. 1a, the resulting Raman spectra of the two phenotypes are extremely similar, with barely any differentiating features that can be identified by eye. Even multivariate methods, such as principal component analysis (PCA) do not show any clear separation on the most representative score plots (see Supplementary Fig. S4). On the other hand, it is possible to generate a model based on LASSO to discriminate between close cellular phenotypes^31,32^. Based on this approach, a model trained with 80% of the data can efficiently distinguish Tconv and Treg with an accuracy of 78.3% on the remaining 20% test data. The performance is illustrated in Fig. 1b with the receiver operating characteristic (ROC) curves of both training and testing data, also demonstrating good stability of the model.

As it has been shown that activation can have a significant influence on the Raman spectra emitted by T cells^30,31^, we first assessed the ability of our approach to distinguish Tconv and Tregs without the influence of activation. We therefore sorted cells based on their activation state that distinguishes naive and effector cells (see Supplementary Fig. S3 for purity plots), and compared the performance of classification models trained exclusively on naive cells and on the overall population (naive and effector cells together) as in Fig. 1b. Detailed results are given as confusion matrices in Supplementary Table S1, showing that while the training performance decreases slightly when considering the overall population versus training exclusively with naive cells (83.2% and 81.4% for naive and full models, respectively), the performance is essentially identical with test data (78.25% and 77.9%, respectively), which shows that there is less cell variability when considering only naive cells, leading to overfitting, and that accurate classification can be also done in the presence of effector cells.

### Tconv and Tregs are identified by protein structure and amino-acids residues

**Figure 2:**
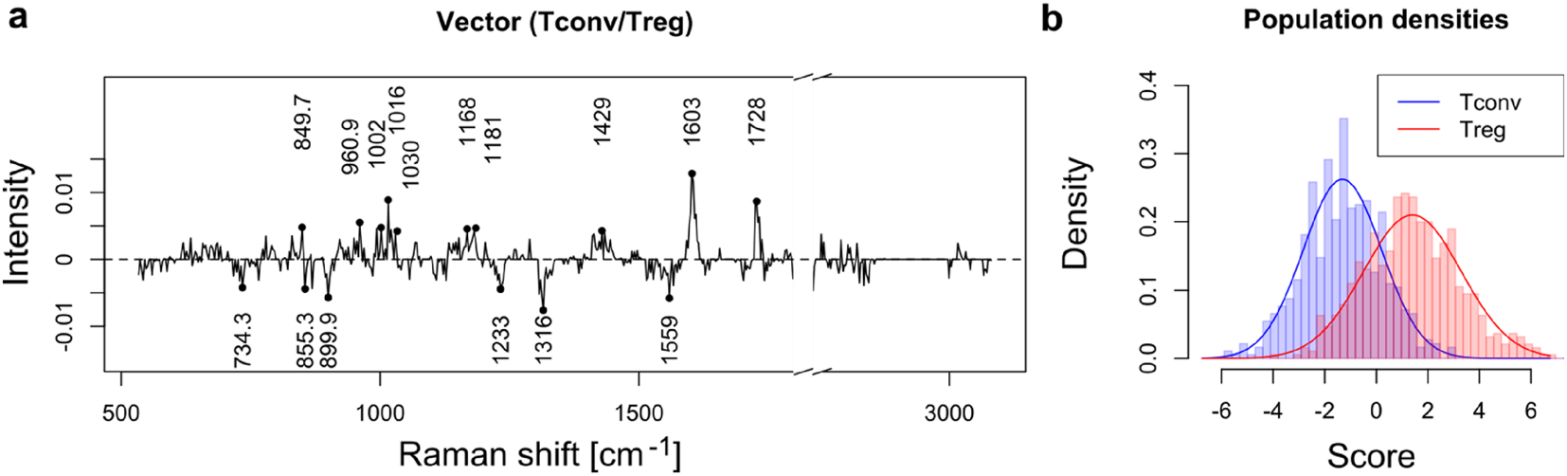
(**a**) Separation vector of the model separating murine Tconv and Treg cells. (**b**) Resulting population density, corresponding to the classification accuracy displayed in Fig. 1b.

LASSO also provides a discrimination vector that allows the identification of the main bands that are contributing to the separation. The vector between murine Tconv and Treg cells is shown in Fig. 2a, where multiple regions contain non-zero values (356 of 643 available variables). While several parts do not provide very clear features, especially in the low wave number region, there are still some clear bands that contribute to the separation. The population distribution resulting from this vector is shown in Fig. 2b for the test data of Fig. 1b.

In particular, multiple amide III bands related to protein structure are present in the negative features that identify Tconv, which can be attributed to β-sheet (1233 cm^−1^ region) and α-helix (1316 cm^−1^ region)^34^. Several bands can be related to tryptophan, in the amide II region (1559 cm^−1^, indole ring^35^ and the ring breathing mode (734 and 855 cm^−1^ regions^36^).

In the positive bands that identify Tregs, several bands related to amino-acids residues can be identified, especially phenylalanine through phenyl ring bands (1603 cm^−1^, 1008/1034 cm^−1^ ^34^) and indole ring bands (1181 cm^−1^ ^36^), although some of these peaks can also be attributed to tyrosine. Remaining positive bands seem to be more generic, and could be attributed to carboxylate ester group, with stretch bands (1410 cm^−1^ ^35^) associated with CH stretching (1449 cm^−1^), and C=O stretch around 1735 cm^−1^ ^37^.

### Raman spectroscopy can identify human Tregs

**Figure 3:**
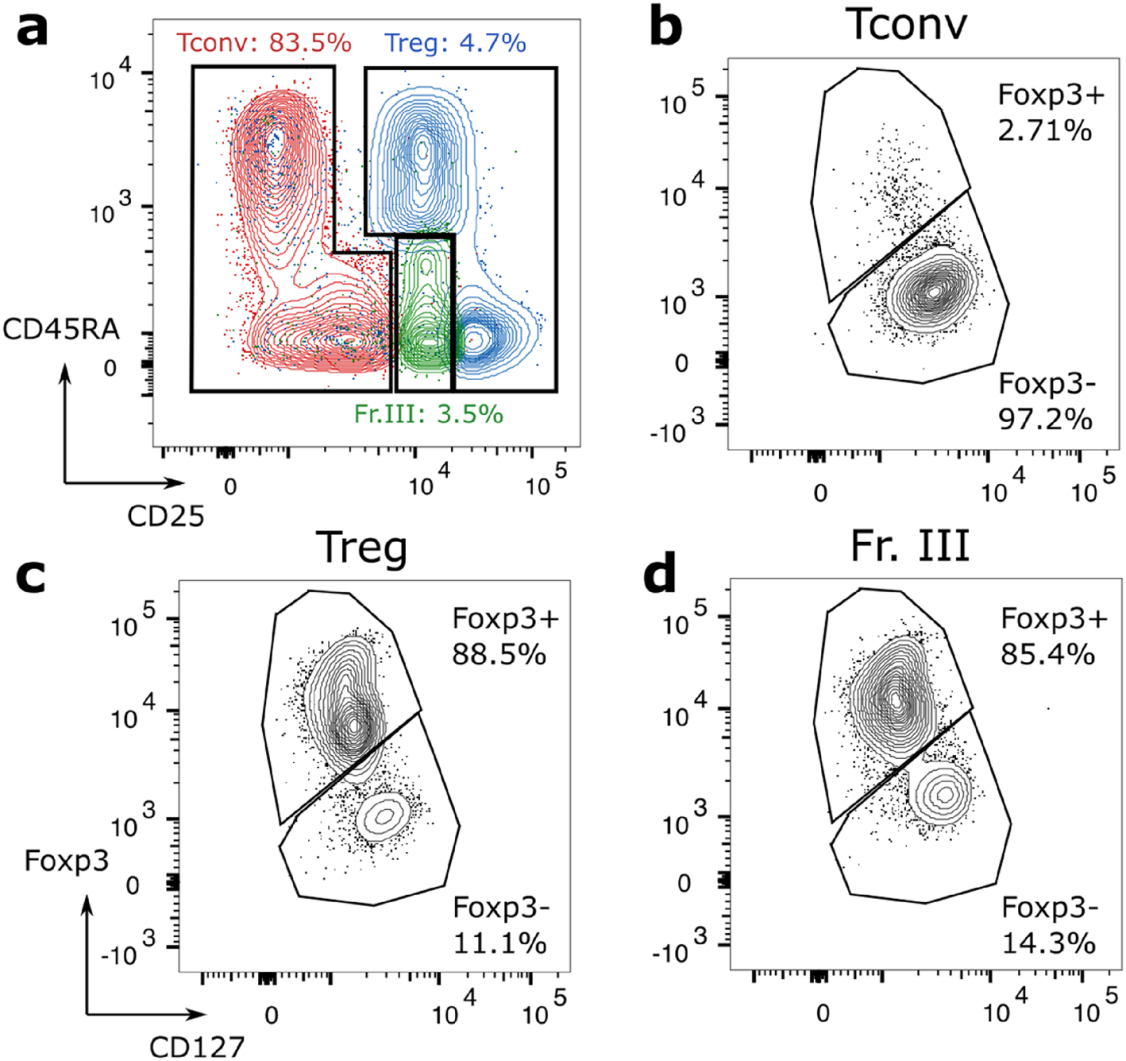
(**a**) Gating strategy of human T cells with CD25/CD45RA markers. Shown populations are sorted cells, showing the purity of each sample, and percentages are ratios during initial sorting. (**b**–**d**) Representative purity of each subpopulation (Tconv, Treg, Fr. III), validated after sorting with CD127/Foxp3 expression.

Having shown the capability of our method on murine cells with a Foxp3 reporter, we then validated it on unaltered human cells. In this preliminary study, we used the blood of 3 donors, each measured on multiple days. Cells were sorted based on their CD25 and CD45RA expression, used as a proxy of Foxp3 and an indicator of activation, respectively (refer to Fig. S2 for details). Typical ratios of the sorted populations are indicated in Fig. 3a. As it was previously demonstrated that reliable models can be generated regardless of the activation state, naive and effector Tconv and Tregs are collected together to ensure a sufficient throughput during sorting, and fraction III (Fr. III)—a commonly denoted subpopulation characterized by its CD25^*mid*^/CD45RA^-^ expression^13^—was also collected separately. As Fr. III is commonly considered to contain a mix of Tconv and Tregs, it is not considered in the initial classification.

The purity of the extraction was then validated on a subset of cells not used for Raman measurements, by checking their CD127/Foxp3 expression (see Fig. 3b–d). This validation shows that the purity can greatly vary between days and donors, and is typically within 87–97% for Tconv, and 73–92% for Tregs. Similarly, the Foxp3 expression in cells in the Fr. III group is highly dependent on the donor, where some samples could reach up to 90% Foxp3^+^ cells, while others could be as low as only 62%.

**Figure 4:**
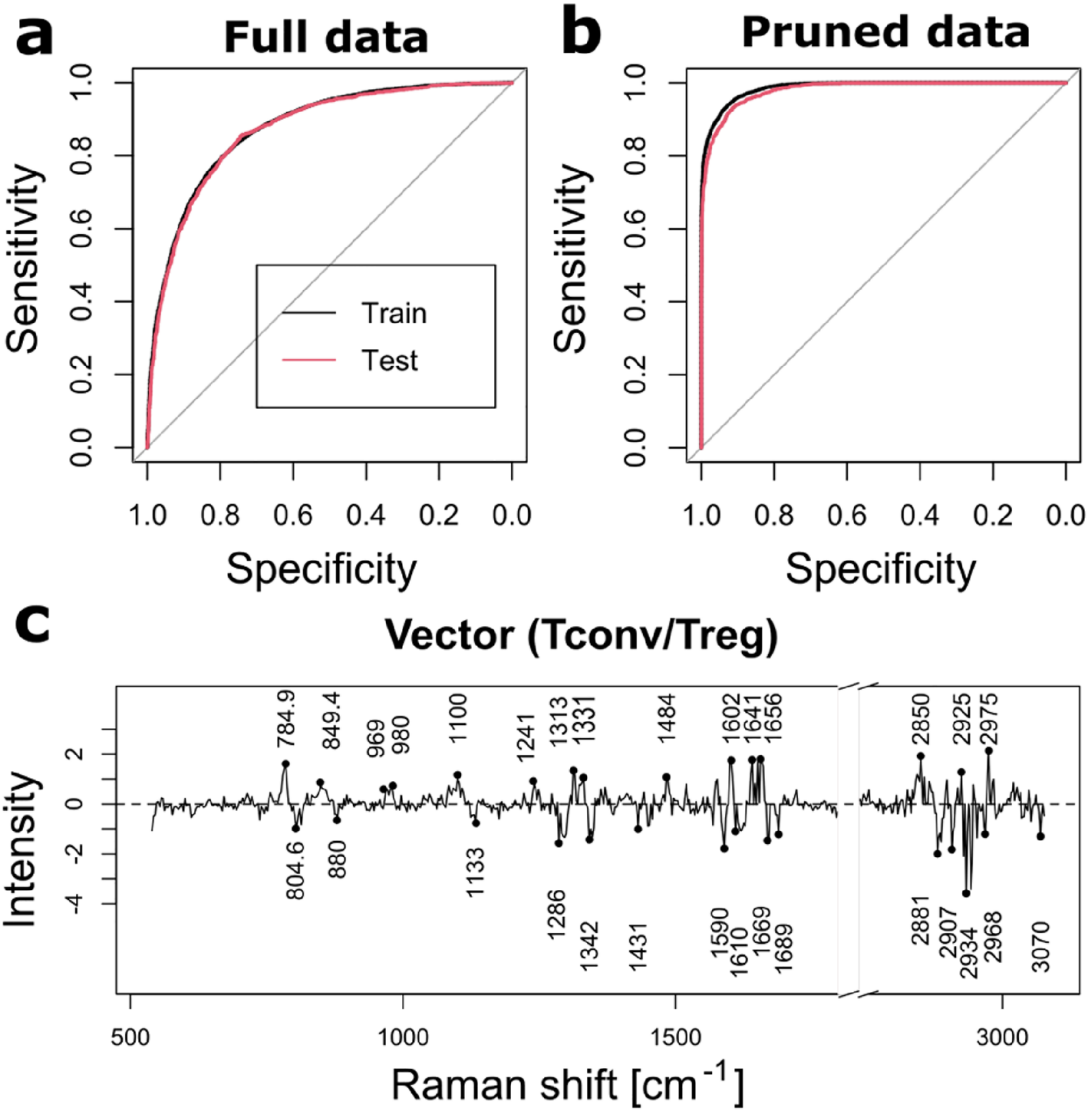
Classification performance for human Tconv/Treg. ROC curves of models based on (**a**) full data (N = 21,655) and (**b**) pruned data (N = 18,079). AUC: 0.8718 and 0.9806, respectively. (**c**) Separation vector identifying human Treg cells of the model based on pruned data.

The overall data is distributed on 3 donors and 9 batches of experiments (N = 21,655). The first model, using 20% of randomly sampled data for testing that was not used during training, could reach around 79.4% of overall accuracy (see Fig. 4a). While the performance is comparable to the model based on murine cells, purity is an important issue when employing machine learning methods, as a low purity essentially corresponds to wrongly labeled cells that corrupt the model and can greatly reduce accuracy.

We therefore treated the data to remove these possibly mislabeled cells, by employing a recently proposed approach of data pruning by ‘confident learning’ (CL), which removes samples that have a label with a low probability of being correct^38^. Applying this procedure to our data set uniformly removes 13.6% of the samples, which interestingly is within the expected sorting purity of Tregs. However, this removal ratio is constant over both phenotypes, so that a disproportionate amount of Tconv may be removed, considering that Tconv are 2 to 5 times higher in concentration compared to Tregs.

A model generated purely on pruned data (with 20% used for test) reaches an accuracy of 92%, with very good stability between training and test sets (see Fig. 4b). The resulting vector, displayed in Fig. 4c is however more complex than in the case of the murine model, with 560 non-zero variables. Clear features are also much harder to identify, and surprisingly, the main ones appear quite different from the peaks that were identified in the case of murine cells, although some similarities in terms of contributing molecular species may be identified. For example, as before, some negative bands can be attributed to protein structure, such as amide III α-helix (1340 cm^−1^ ^35^, 1286 cm^−1^ ^36^) or amide I (1619, 1669 cm^−1^ ^37^) although several other bands are challenging to identify. On the other hand, the main positive bands that can be identified seem to be related to DNA/RNA, either through cytosine/uracil ring breathing (785 cm^−1^ ^39^) or PO_2_ stretching^36^. Even if these assignments must be considered with care considering the noise levels of the separation vector, it seems to indicate that the molecular species responsible for the discrimination in case of human cells are different from the murine Tregs, or that they contribute in different ratios.

### Raman models are consistent with donor variability

**Figure 5:**
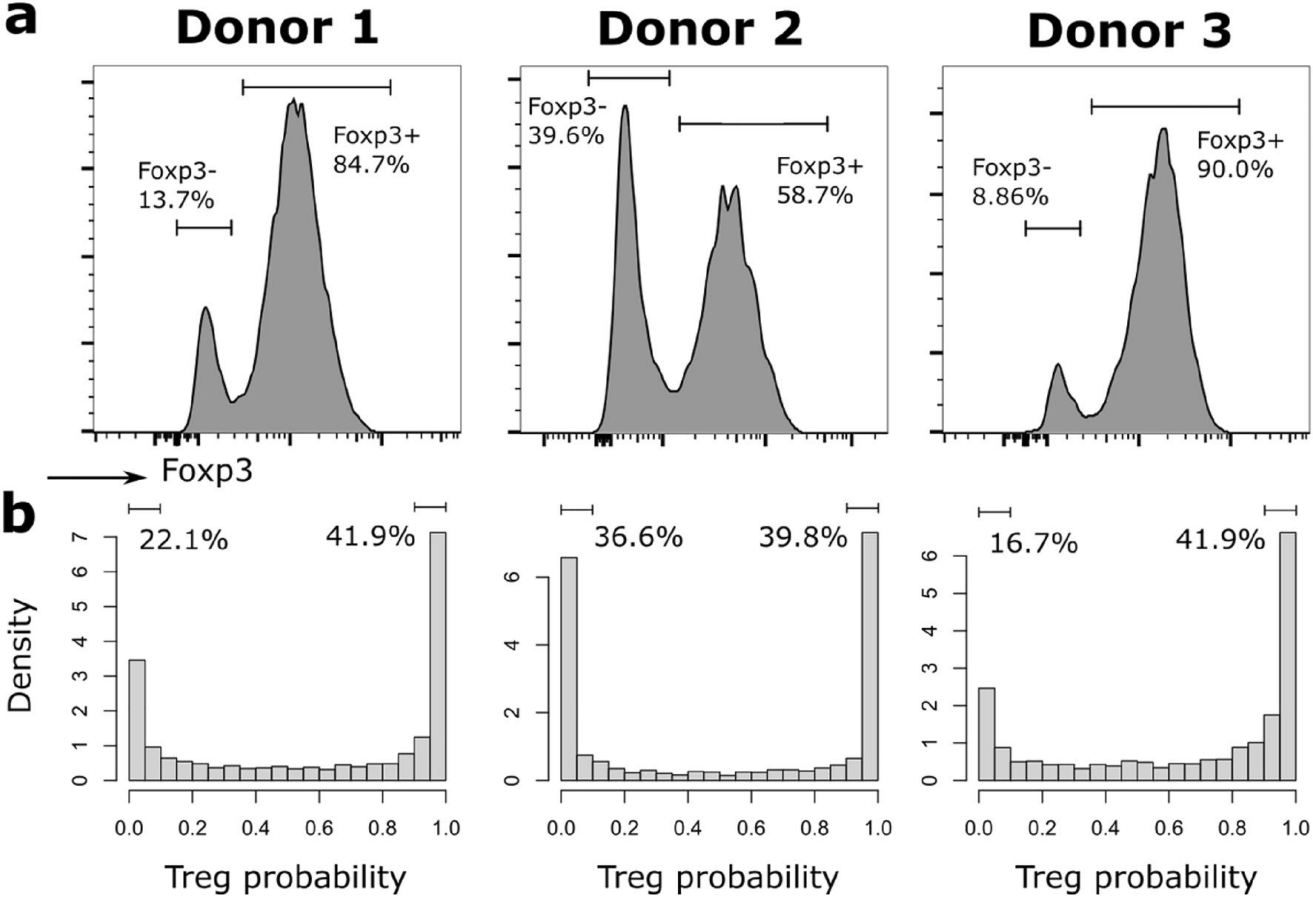
Distribution of cells sub-population in fraction III, shown separately by donor. (**a**) Foxp3 expression. (**b**) Probability of being a Treg based on the pruned label-free model displayed in Fig. 4b, cell ratios for the 10% and 90% probabilities are indicated for each donor.

To further validate the adequacy of our model based on pruned data as shown in Fig. 4b, we applied it to all Fr. III cells (without pruning, N = 10,410), and analyzed the results separately by donor as cell distributions can widely vary, as shown in Fig. 5a, where the Foxp3 expression of Fr. III cells is shown for each donor. The results are displayed as probabilities of being Tregs (see Fig. 5b), where it is possible to see that they are heavily skewed towards extreme values, implying that Fr. III cells have a very high probability to be either Tconv or Treg, which is consistent with literature^13^. The ratios, indicated as proportions of cells with *p* < 10% and *p* > 90%, are also very comparable to Foxp3 expression. In particular, cells from donor 2 appear to have a much higher Foxp3^-^ population, which is consistent with a higher proportion of cells being scored with a very low probability of being Tregs. These results indicate a strong correlation between the scores derived from the Raman models and Foxp3 expression, here validated independently on the Fr. III population, which was not employed during training.

### Raman models can detect Tregs from independent donors

**Figure 6:**
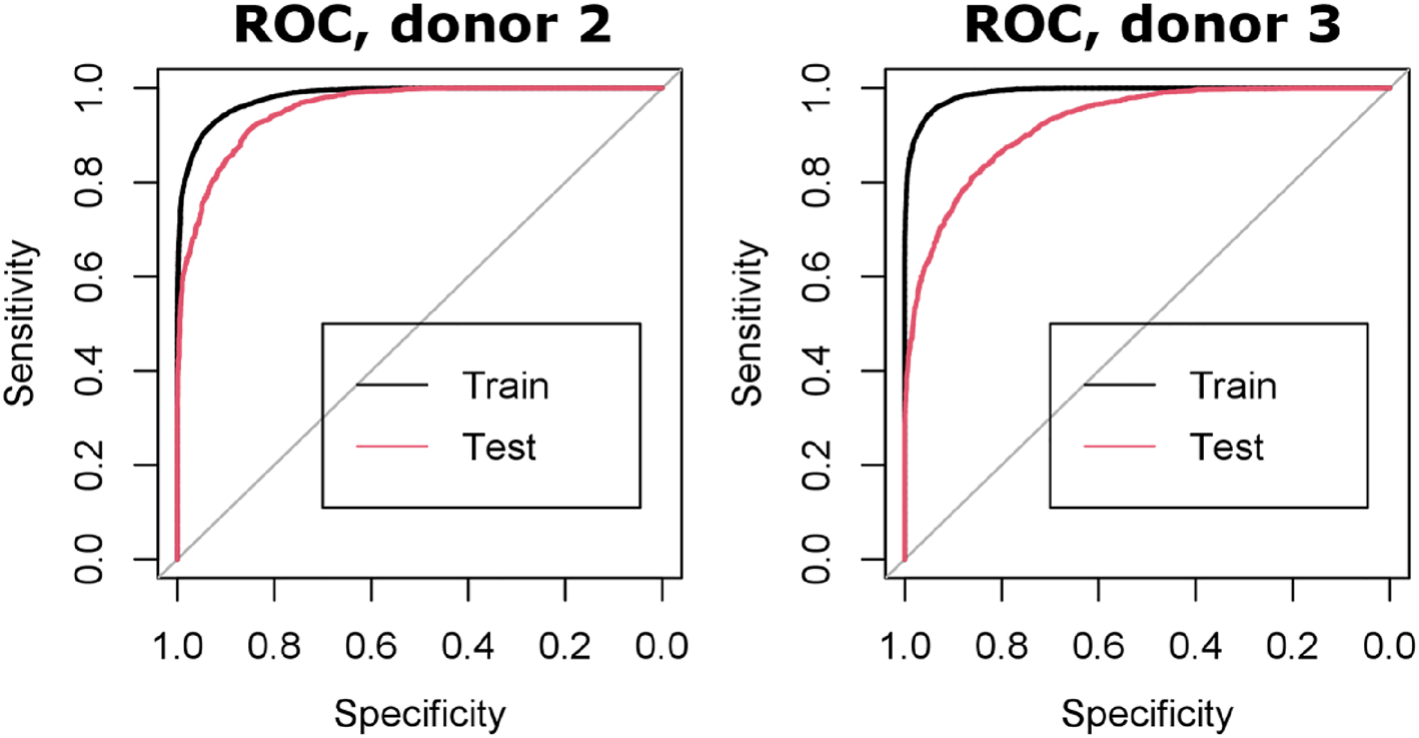
ROC curves illustrating classification performance of human Tconv/Treg cells with independent donors as test data (27.1% and 27.64% of total data for donor 2 and 3, respectively.) AUC: 0.9567 and 0.9243 for donor 2 and 3, respectively.

One key point for the applicability of our proposed approach is the ability of detecting Tregs from a new patient sample that is fully independent from data that was used to generate the model. To assess the ability of our method to accomplish this, we generated models derived from the pruned data of two of the available donors, and tested it on the remaining one. In the first case, we used a model generated with donors 1 and 3 to detect Tregs from the cells of donor 2 (27.1% of total data). In the second, a model from donors 1 and 2 was used on cells from donor 3 (27.64% of total data). In both cases, very high accuracies are obtained during training (> 90%), as shown in Fig. 6. Performance of test data from an independent donor is slightly lower, showing that predictions are more difficult than in the previous cases where test data was randomly sampled from the whole data set. This is expected as the model with random sampling was exposed to a wider variety of samples during training. The performance is still comparatively quite high, with 87.4% and 82.4% of accuracy for tests on donor 2 and 3, respectively, which demonstrates the ability of our models to accurately distinguish Tregs. The performance of our approach is also in the same range as typical purities when sorting cells based on CD25/CD45RA surface markers. While these results are still quite preliminary with data based on only 2 donors to predict another, they are especially encouraging as we can expect the performance to increase when more donors are included in the model, which should improve the transferability of the predictions.

## Discussion

This study examines regulatory T cells, a relatively rare cell type known for its clinical significance in preventing autoimmune diseases, for example. They are known to be very similar to conventional CD4^+^ cells, with no specific surface markers, so that only the intracellular marker Foxp3 can conclusively distinguish Tregs. Though surrogate surface markers also have potential to identify Tregs, ambiguity remains with activated Tconv cells that also express these markers. A non-invasive method such as the one described here could therefore greatly improve the reliability of detection of unaltered live Treg cells.

It was shown that the Raman spectral differences between Tconv and Tregs are not detectable visually or by conventional unsupervised methods, showing that these two phenotypes are not trivial to distinguish even with spectroscopy. Nevertheless, it is possible to generate reliable statistical models that can distinguish murine Tconv and Tregs with reasonable accuracy (> 78%), while also providing a separation vector with strong identifiable bands. These bands, which are correlated with Foxp3 expression through the separation of Tconv and Tregs, can be linked with specific molecular structures, show that the identification is mostly coming from intracellular protein structure and amino-acids concentrations, as Raman spectroscopy retrieves an endogenous signal representative of all intracellular molecules.

A similar approach made it possible to then distinguish human Tconv and Tregs extracted from PBMCs, with comparable accuracy to murine cells, despite the addition of the donor to donor variability. However, to train the model, classes that had been sorted based on surrogate markers had to be employed, implying that the labels assigned to measured cells used for training may be erroneous due to sample impurity, which can greatly affect the performance of statistical models. We therefore used a recently proposed approach of data pruning to improve the quality of training data sets for machine learning. The problem of cellular impurities that affect the model accuracy is equivalent in terms of data analysis to mislabeled samples, so that these methods can be directly employed.

Interestingly, data pruning removes an amount of data which is comparable with the purity level of sample as externally validated by FACS with intracellular staining, showing some level of consistency for this approach. The models trained with that method reach an accuracy over 90%, despite the day-to-day and donor-to-donor variations. Furthermore, these models applied to the Fraction III population, considered to be a mix of Tconv and Tregs sub-populations, display results that are highly consistent with external validation. The label-free prediction applied to the Fr. III of each donor is consistent with the Foxp3 expression of these populations, further confirming the biological relevance of that model.

Finally, one key point to allow usage of this method for practical application is to demonstrate the applicability of the model not only to new samples (cells) but to fully independent new donors, i.e. that have not been used at all during training stage. Even in the case of the small data set of this preliminary study (3 donors), it was possible to demonstrate that the data from 2 donors can be used to classify cells from the last donor with satisfying accuracy (> 80%), which is comparable with typical sorting purity based on surrogate surface markers.

This ability demonstrates the potential of this approach for the detection of Tregs within fully independent populations. Furthermore, one should note that the confidence rate of the classification can be adjusted. All accuracy values reported here have been obtained with a threshold at 0.5, while it is also possible to gain sensitivity for higher confidence, of course at the cost of specificity, as illustrated by ROC curves.

One significant limitation of the method employed here compared to labelled approaches is throughput, where the system currently uses automated sequential detection that can reach around 1000 cells/hour, which is not sufficient for practical use cases that can require 10^6^–10^9^ cells depending on the application. Solutions that combine for instance shorter exposure time, where models based on larger data sets could better cope with noise, or parallel detection could be considered to reach higher throughput. This implies, however that while the detection of unmodified Tregs from a CD4^+^ T cell population is possible, significant development will still be required for applications that require higher cell throughput.

Finally, for advanced and clinical applications, this noninvasive approach could also be combined with fluidics methods to allow Raman-based sorting and purification, as it was recently demonstrated for spontaneous Raman applied to bacteria^40^. This would also enable paired comparisons, i.e. Foxp3 expression and Raman evaluation on the same cells for more accurate validation of this method. This would also allow the confirmation of unbiased detection through paired validation, while opening new possibilities such as repeated measurements through time on live cells, as it has been shown that Foxp3 expression can occur transiently also in conventional T cells^41,42^.

## Materials and methods

### Murine samples

This study followed ARRIVE guidelines on the use of experimental animals^43^. All animal experiments were conducted with the approval of the Animal Research Committee of the Research Institute for Microbial Diseases in Osaka University, Japan, and in accordance with the guidelines of the Animal Care and Use Committee of Osaka University. All mice used in this study were of the C57BL/6J genetic background, maintained under specific pathogen-free conditions in the experimental animal facilities at the Immunology Frontier Research Center, Osaka University. Foxp3^*tm1(CD2/CD52)Shori*^ (Foxp3-hCD2) mice, which express human CD2 as a cell surface reporter for Foxp3, were provided as a courtesy by Prof. Shohei Hori, University of Tokyo.

### Human samples

Peripheral blood samples were obtained from healthy volunteer donors. All donors provided written informed consent before sampling, according to the Declaration of Helsinki. This study was approved by the Osaka University Research Ethics Committee (http://www.osaka-u.ac.jp/en/research/iinkai/moral/index.html) (Osaka, Japan). All donors were healthy volunteers, in the age range of 25–45.

### Cell isolation

PBMC are isolated from donor blood samples using Ficoll-Paque (GE-Healthcare) density gradient and LeucoSEP separation tubes (Greiner Bio-One). Spleens are collected from Foxp3-hCD2 mice, and single-cell suspensions are obtained by mechanical homogenization through 40 μm cell strainers (Corning) and treatment with RBC lysis buffer (Sigma).

CD4^+^ T cells are isolated by negative selection using a CD4^+^ T Cell Isolation Kit (human or mouse as appropriate) and an autoMACS Pro Separator or MACS Cell Separation LS Columns (Miltenyi Biotec). Collected mouse CD4^+^ T cells are stained with anti-human CD2 Brilliant Violet 421 (RPA-2.10), anti-CD62L Brilliant Violet 711 (MEL-14) and anti-CD44 Alexa Fluor 700 (IM7); human CD4^+^ T cells are stained with anti-CD4 Alexa Fluor 700 (RPA-T4), anti-CD25 Brilliant Violet 421 (BC96), and anti-CD45RA Brilliant Violet 711 (HI100). Cell sorting is performed on a BD FACSAria III, and sorted cells are collected in RPMI 1640 medium without phenol red (Agilent) supplemented with 10% FBS.

As described in Miyara et al.^13^, mouse Tregs are defined as hCD2^+^, and Tconv as hCD2^-^; activated cells are defined as CD44^*hi*^/CD62L^*lo*^, and naive cells as CD44^*lo*^/CD62L^*hi*^; human Tconv (combined Fractions IV, V and VI), Treg (combined Fractions I and II) and Fraction III cells are collected based on their CD45RA/CD25 expression.

Sorting purity is confirmed by further acquisition of the sorted samples. For population purity determination, aliquots of sorted samples are fixed and permeabilized with the Foxp3/Transcription Factor Staining Buffer Set (eBioscience), then stained with anti-Foxp3 PE (mouse: FJK-16s, human: 236A/E7; both from eBioscience). Additionally, human cells are stained with anti-CD127 FITC (A019D5) and mouse cells with anti-CD4 APC (RM4-5). All antibodies were purchased from BioLegend unless otherwise stated and used at a dilution of 1:200.

### Raman measurements

Single-cell suspensions are plated in 4-well micro-inserts (Ibidi) fixed on 35 mm quartz dishes (Matsunami). Raman spectra acquisition is then performed with a system optimized and automated for single-cell measurements as described previously^30,44,45^.

Briefly, cells are imaged with a quantitative phase microscopy (QPM) system based on off-axis digital holography^46^. The QPM images are reconstructed and segmented^47,48^ to identify cells in the field of view. Cells are then illuminated with a 532 nm laser focused with a 60× objective (NA 1.27), with a local power at the sample of 487.5 mW/μm^2^. The spot is rapidly scanned within a region that covers approximately 60–90% of the cell body to retrieve an optically averaged signal, representative of the overall cellular content^45^. The back-scattered light is collected by the objective, dispersed by a 500 mm Czerny-Turner spectrometer with a 300 lp/mm grating and recorded with a sCMOS camera with an acquisition time of 3 s per cell. The fully automated system can typically record 1000 cells/hour.

### Data analysis

Raman data pre-processing is done with custom scripts in Matlab (Mathworks), where spectra are first baseline corrected with cubic spline interpolation, and cosmic rays are removed through median filtering. Spectral values are then adjusted by interpolating the spectra on a grid defined by a calibration spectrum of pure ethanol measured each day. The silent region (1800–2700 cm^−1^) is then removed.

Data processing is then performed with the *R* program^49^. Principal component analysis is done with built-in functions, and outliers are manually removed by identifying them in score plots. Receiver operating characteristic (ROC) calculations and logistic regression, regularized with LASSO, are performed with the *pROC*^50^, and *glmnet*^51^ packages, respectively. Implementation details for Raman-based classification were given previously^32^. Briefly, processed data is employed directly for model training. Test data is always independent data that was not seen by the model during training to provide unbiased prediction performance. Confident learning^38^ is performed with the *cleanlab* package in Python.

### Supplementary Information

Supplementary Information.

## Data Availability

All data needed to evaluate the conclusions in the paper are present in the paper and/or the Supplementary Materials. The raw data and datasets acquired and generated during the current study are available from the corresponding author on reasonable request.

## References

[1] Kaech SM, Wherry EJ, Ahmed R (2002). Effector and memory T-cell differentiation: Implications for vaccine development. Nat. Rev. Immunol..

[2] Sakaguchi S, Fukuma K, Kuribayashi K, Masuda T (1985). Organ-specific autoimmune diseases induced in mice by elimination of T cell subset. I. Evidence for the active participation of T cells in natural self-tolerance; deficit of a T cell subset as a possible cause of autoimmune disease. J. Exp. Med..

[3] Powrie F, Mason D (1990). OX-22high CD4^+^ T cells induce wasting disease with multiple organ pathology: Prevention by the OX-22low subset. J. Exp. Med..

[4] Sakaguchi S, Sakaguchi N, Asano M, Itoh M, Toda M (1995). Immunologic self-tolerance maintained by activated T cells expressing IL-2 receptor alpha-chains (CD25). Breakdown of a single mechanism of self-tolerance causes various autoimmune diseases. J. Immunol..

[5] Bennett CL (2001). The immune dysregulation, polyendocrinopathy, enteropathy, X-linked syndrome (IPEX) is caused by mutations of FOXP3. Nat. Genet..

[6] Brunkow ME (2001). Disruption of a new forkhead/winged-helix protein, scurfin, results in the fatal lymphoproliferative disorder of the scurfy mouse. Nat. Genet..

[7] Farh KK-H (2014). Genetic and epigenetic fine mapping of causal autoimmune disease variants. Nature.

[8] Ohkura N (2020). Regulatory T cell-specific epigenomic region variants are a key determinant of susceptibility to common autoimmune diseases. Immunity.

[9] Ferreira LMR, Muller YD, Bluestone JA, Tang Q (2019). Next-generation regulatory T cell therapy. Nat. Rev. Drug Discov..

[10] Sakaguchi S (2021). Taking regulatory T cells into medicine. J. Exp. Med..

[11] Hori S, Nomura T, Sakaguchi S (2003). Control of regulatory T cell development by the transcription factor Foxp3. Science.

[12] Fontenot JD, Gavin MA, Rudensky AY (2003). Foxp3 programs the development and function of CD4^+^CD25^+^ regulatory T cells. Nat. Immunol..

[13] Miyara M (2009). Functional delineation and differentiation dynamics of human CD4^+^ T cells expressing the Foxp3 transcription factor. Immunity.

[14] Santegoets SJAM (2015). Monitoring regulatory T cells in clinical samples: consensus on an essential marker set and gating strategy for regulatory T cell analysis by flow cytometry. Cancer Immunol. Immunother..

[15] Seddiki N (2006). Expression of interleukin (IL)-2 and IL-7 receptors discriminates between human regulatory and activated T cells. J. Exp. Med..

[16] Liu W (2006). CD127 expression inversely correlates with FoxP3 and suppressive function of human CD4^+^ T reg cells. J. Exp. Med..

[17] Shimizu J, Yamazaki S, Takahashi T, Ishida Y, Sakaguchi S (2002). Stimulation of CD25^+^CD4^+^ regulatory T cells through GITR breaks immunological self-tolerance. Nat. Immunol..

[18] McHugh RS (2002). CD4^+^CD25^+^ immunoregulatory t cells: gene expression analysis reveals a functional role for the glucocorticoid-induced TNF receptor. Immunity.

[19] Goding JW (1982). Biological effects of antibodies to lymphocyte surface receptors. Springer Semin. Immunopathol..

[20] Andrä I (2020). An evaluation of T-cell functionality after flow cytometry sorting revealed p38 MAPK activation. Cytom. Part A.

[21] Shipp DW, Sinjab F, Notingher I (2017). Raman spectroscopy: techniques and applications in the life sciences. Adv. Opt. Photonics.

[22] Pahlow S, Meisel S, Cialla-May D, Weber K, Rösch P, Popp J (2015). Isolation and identification of bacteria by means of Raman spectroscopy. Adv. Drug Deliv. Rev..

[23] Ho C-S (2019). Rapid identification of pathogenic bacteria using Raman spectroscopy and deep learning. Nat. Commun..

[24] Lloyd GR (2013). Discrimination between benign, primary and secondary malignancies in lymph nodes from the head and neck utilising Raman spectroscopy and multivariate analysis. Analyst.

[25] Kong K (2013). Diagnosis of tumors during tissue-conserving surgery with integrated autofluorescence and Raman scattering microscopy. Proc. Natl. Acad. Sci. USA.

[26] Okada M (2012). Label-free Raman observation of cytochrome c dynamics during apoptosis. Proc. Natl. Acad. Sci. USA.

[27] Pavillon N, Hobro AJ, Akira S, Smith NI (2018). Noninvasive detection of macrophage activation with single-cell resolution through machine learning. Proc. Natl. Acad. Sci. USA.

[28] Schie IW (2018). High-throughput screening raman spectroscopy platform for label-free cellomics. Anal. Chem..

[29] Pavillon N, Smith NI (2019). Immune cell type, cell activation, and single cell heterogeneity revealed by label-free optical methods. Sci. Rep..

[30] Ichimura T (2016). Non-label immune cell state prediction using Raman spectroscopy. Sci. Rep..

[31] Pavillon N, Smith NI (2023). Non-invasive monitoring of T cells differentiation through Raman spectroscopy. Sci. Rep..

[32] Pavillon N, Smith NI (2021). Deriving accurate molecular indicators of protein synthesis through Raman-based sparse classification. Analyst.

[33] Komatsu N, Mariotti-Ferrandiz ME, Wang Y, Malissen B, Waldmann H, Hori S (2009). Heterogeneity of natural Foxp3 T cells: A committed regulatory T-cell lineage and an uncommitted minor population retaining plasticity. Proc. Natl. Acad. Sci. USA.

[34] Ellis DI, Cowcher DP, Ashton L, O’Hagan S, Goodacre R (2013). Illuminating disease and enlightening biomedicine: Raman spectroscopy as a diagnostic tool. Analyst.

[35] Ashton L, Blanch EW (2010). pH-induced conformational transitions in α-lactalbumin investigated with two-dimensional Raman correlation variance plots and moving windows. J. Mol. Struct..

[36] Notingher I, Verrier S, Haque S, Polak JM, Hench LL (2003). Spectroscopic study of human lung epithelial cells (A549) in culture: Living cells versus dead cells. Biopolymers.

[37] Maquelin K (2002). Identification of medically relevant microorganisms by vibrational spectroscopy. J. Microbiol. Methods.

[38] Northcutt C, Jiang L, Chuang I (2021). Confident learning: Estimating uncertainty in dataset labels. J. Artif. Intell. Res..

[39] Byrne, H., Sockalingum, G. & Stone, N. Raman Microscopy: Complement or Competitor?” *RSC Analytical Spectroscopy Series* (2011).

[40] Lee KS (2019). An automated Raman-based platform for the sorting of live cells by functional properties. Nat. Microbiol..

[41] Gavin MA (2006). Single-cell analysis of normal and FOXP3-mutant human T cells: FOXP3 expression without regulatory T cell development. Proc. Natl. Acad. Sci. USA.

[42] Miyao T (2012). Plasticity of Foxp3+ t cells reflects promiscuous Foxp3 expression in conventional T cells but not reprogramming of regulatory T cells. Immunity.

[43] Kilkenny C, Browne JB, Cuthill IC, Emerson M, Altman DG (2010). Improving bioscience research reporting: The ARRIVE guidelines for reporting animal research. PLoS Biol..

[44] Pavillon N, Hobro AJ, Smith NI (2013). Cell optical density and molecular composition revealed by simultaneous multimodal label-free imaging. Biophys. J..

[45] Pavillon N, Smith NI (2015). Maximizing throughput in label-free microspectroscopy with hybrid Raman imaging. J. Biomed. Opt..

[46] Cuche E, Marquet P, Depeursinge C (1999). Simultaneous amplitude–contrast and quantitative phase–contrast microscopy by numerical reconstruction of Fresnel off–axis holograms. Appl. Opt..

[47] Colomb T, Kühn J, Charrière F, Depeursinge C, Marquet P, Aspert N (2006). Total aberrations compensation in digital holographic microscopy with a reference conjugated hologram. Opt. Express.

[48] Stringer C, Wang T, Michaelos M, Pachitariu M (2020). Cellpose: a generalist algorithm for cellular segmentation. Nat. Methods.

[49] R Core Team. *R: A Language and Environment for Statistical Computing*. R Foundation for Statistical Computing, Vienna, Austria (2016).

[50] Robin X (2011). pROC: An open-source package for R and S+ to analyze and compare ROC curves. BMC Bioinform..

[51] Friedman J, Hastie T, Tibshirani R (2010). Regularization paths for generalized linear models via coordinate descent. J. Stat. Softw..

