# Supplementary material for: Non-invasive detection of regulatory T cells with Raman spectroscopy
Source: Sci Rep. 2024 Jun 18;14:14025. doi: 10.1038/s41598-024-64536-0 (PMC11189440; doi:10.1038/s41598-024-64536-0)
Supplement: Supplementary file 1 — Supplementary Information. [file 41598_2024_64536_MOESM1_ESM.docx]

Supplementary information for

Non-invasive detection of regulatory T cells with Raman spectroscopy

N. Pavillon^1,*^, E. L. Lim^2^, A. Tanaka^2,3^, S. Hori^4^, S. Sakaguchi^2,5^, N. I. Smith^1,6,7*^

Biophotonics Laboratory, Experimental Immunology, Immunology Frontier Research Center (IFReC), ^3^Department of Frontier Research in Tumor Immunology, Graduate School of Medicine, Osaka University, Osaka, Japan.

^4^Laboratory of Immunology and Microbiology, Graduate School of Pharmaceutical Sciences, The University of Tokyo, Tokyo, Japan.

^5^Laboratory of Experimental Immunology, Institute for Life and Medical Sciences, Kyoto University, Kyoto, Japan

^6^Center for Infectious Disease Education and Research (CiDER), ^7^Open and Transdisciplinary Research Institute (OTRI), Osaka University, Suita, Osaka, Japan.

[*n-pavillon@ifrec.osaka-u.ac.jp](mailto:*n-pavillon@ifrec.osaka-u.ac.jp), *[nsmith@ap.eng.osaka-u.ac.jp](mailto:nsmith@ap.eng.osaka-u.ac.jp)

**This file includes:**

Figs. S1 to S4 and Table S1


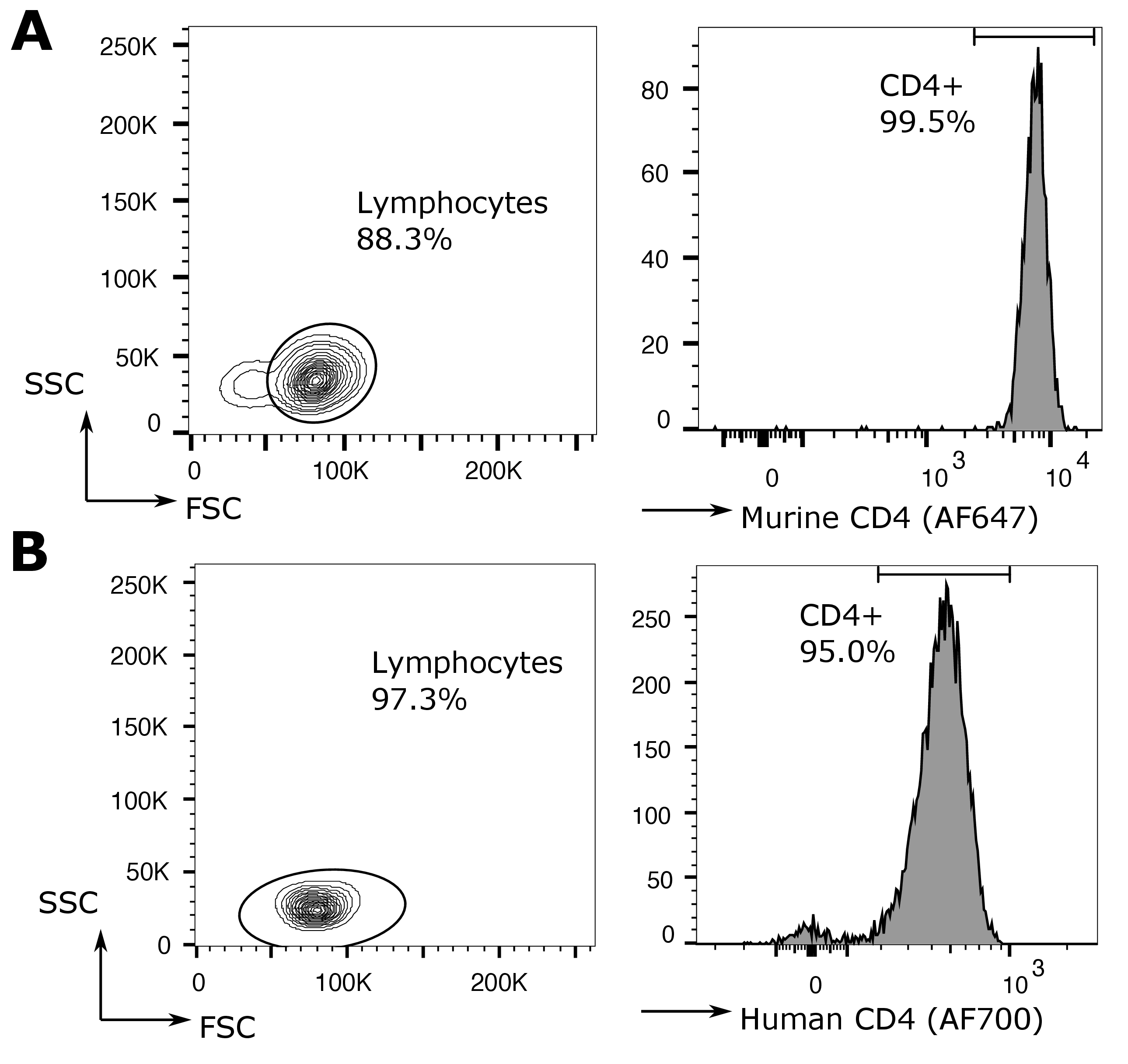


Figure S1: Typical sample purity after MACS sorting for (A) murine lymphocytes (B) humans PBMCs.


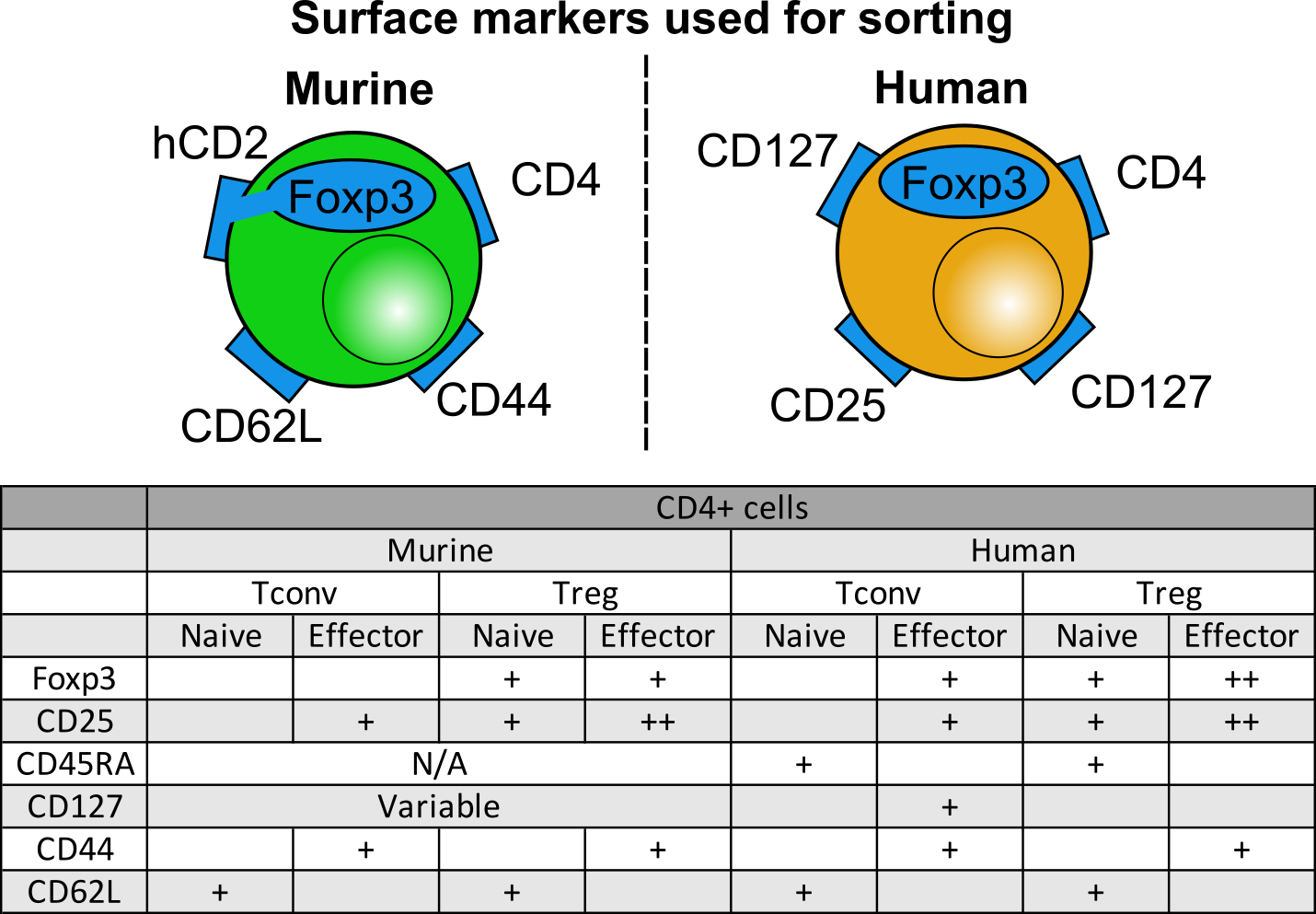


Figure S2: Summary of the markers on CD4^+^ T cells used to distinguish Tconv vs. Treg cells and naive vs effector cells, for both murine and human samples, along with typical expression of the surface markers for the studied cell types. Murine cells possess a surface Foxp3 reporter hCD2.


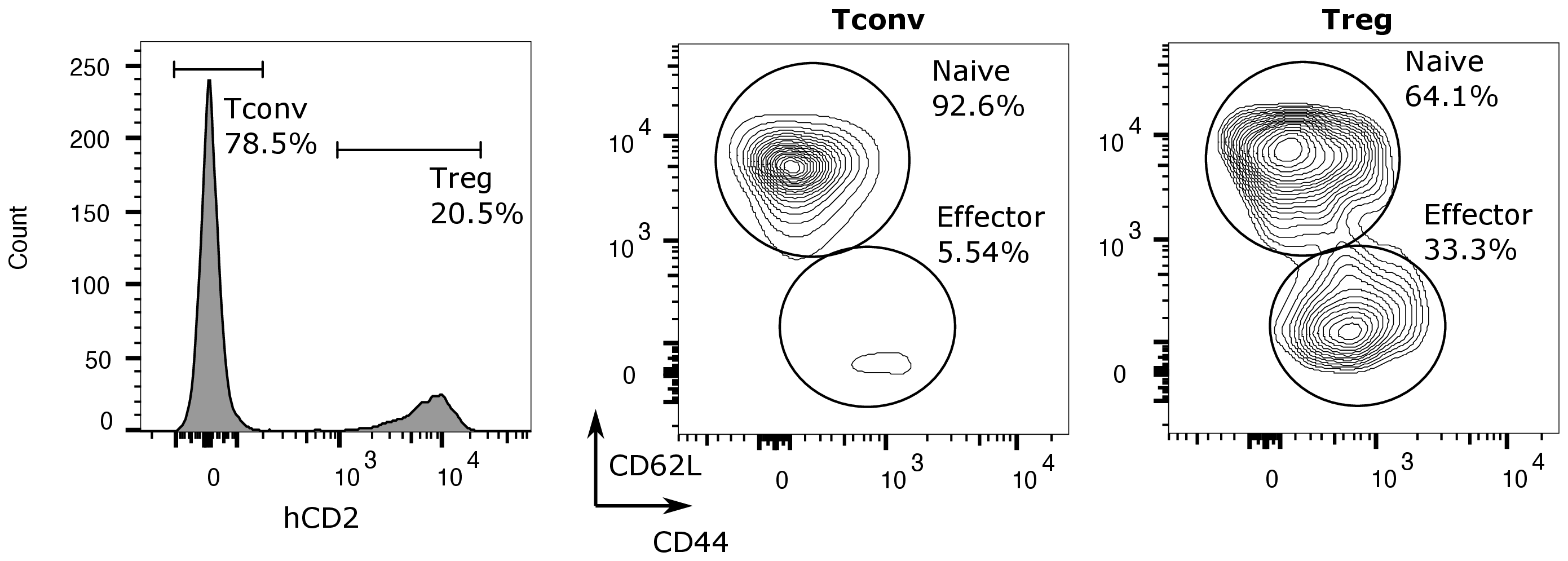


Figure S3: Typical population ratios in murine cells sorting. (left) Tconv/Treg ratio based on Foxp3 reporter. (right) Distribution of naive and effector cells for Tconv and Treg cells, respectively.


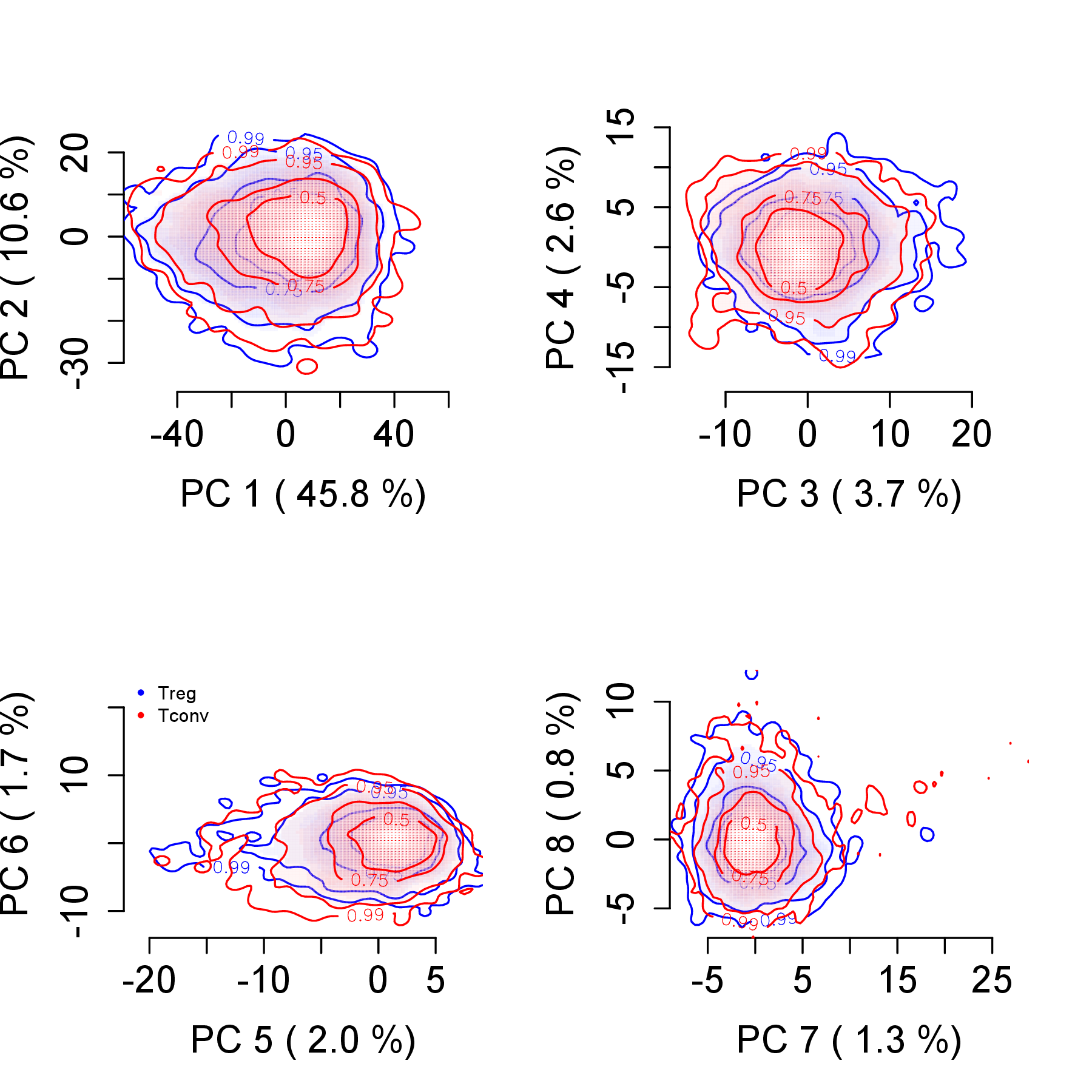


Figure S4: PCA score plots of CD4 murine cells, showing that both Tconv and Tregs have very similar characteristics. Values in parentheses represent percentage of variance for each component.

|  | | Cells (only naive) | | Full (naive + effector) | |
| --- | --- | --- | --- | --- | --- |
|  | | Tconv | Treg | Tconv | Treg |
|  | Tconv | 84.5 | 15.5 | 81.4 | 18.6 |
| Train | Treg | 18.1 | 81.9 | 18.8 | 81.2 |
|  | Tconv | 80.3 | 19.7 | 79.0 | 21.0 |
| Test | Treg | 23.7 | 76.3 | 23.0 | 77.0 |

Table S1: Confusion matrices, comparing models generated purely on resting (naive) cells or the full set, showing similar performances.
